# Feature Selection Method Using Multi-Agent Reinforcement Learning Based on Guide Agents

**DOI:** 10.3390/s23010098

**Published:** 2022-12-22

**Authors:** Minwoo Kim, Jinhee Bae, Bohyun Wang, Hansol Ko, Joon S. Lim

**Affiliations:** 1 Department of Computer Science, Gachon University, Sujeong-gu, Seongnam-si 13557, Gyeonggi-do, Republic of Korea; 2 AI Team, 2nd R&D Center, MEZOO Co., Ltd., Gieopdosi-ro 200, Jijeong-myeon, Wonju-si 26354, Gangwon-do, Republic of Korea; 3 Department of Computer Science, University of Southern California, Los Angeles, CA 90007, USA

**Keywords:** feature selection, guide agents, main agents, multi-agent, reinforcement learning (RL), rewards

## Abstract

In this study, we propose a method to automatically find features from a dataset that are effective for classification or prediction, using a new method called multi-agent reinforcement learning and a guide agent. Each feature of the dataset has one of the main and guide agents, and these agents decide whether to select a feature. Main agents select the optimal features, and guide agents present the criteria for judging the main agents’ actions. After obtaining the main and guide rewards for the features selected by the agents, the main agent that behaves differently from the guide agent updates their Q-values by calculating the learning reward delivered to the main agents. The behavior comparison helps the main agent decide whether its own behavior is correct, without using other algorithms. After performing this process for each episode, the features are finally selected. The feature selection method proposed in this study uses multiple agents, reducing the number of actions each agent can perform and finding optimal features effectively and quickly. Finally, comparative experimental results on multiple datasets show that the proposed method can select effective features for classification and increase classification accuracy.

## 1. Introduction

Various methods and technologies have emerged and, as the cost of collecting data through these methods and technologies decreases, the number of variables that can be used increases exponentially [1,2,3]. To successfully train a machine learning model, it is important to select features that do not adversely affect the prediction of the model and minimize the variation in judgment [4]. In addition, these optimized features positively affect the interpretation of the results and achieve high accuracy [5,6].

Feature selection in the field of machine learning was studied for an exceptionally long time [7]. Feature selection methods include minimum redundancy maximum relevance (mRMR) [8], Relief [9], and a method using a heuristic algorithm, such as the genetic algorithm [10]. Although feature selection methods are mostly fast and efficient, it is difficult to find the best feature subset. In addition, to find the optimal features either the sequential forward search method (SFS), which adds features individually starting with the one with the highest importance, or the sequential backward search (SBS) method, which removes the low-importance features individually from the entire dataset [11], is employed.

In addition, in recent years, a feature selection method, such as Recursive Feature Elimination (RFE) or Recursive Feature Elimination with Cross Validation (RFECV) using a boosting model such as Light Gradient Boosting Model (LightGBM), has been widely used [12,13,14]. These methods are not a big problem when the number of features in a dataset is small, but when this number is large, considerable computation time and computing power are required. Although feature selection methods using heuristic optimization algorithms, such as genetic algorithms, work flexibly on multiple datasets, it is difficult to make detailed selections because features are judged based on a combination of features rather than individually [15,16,17].

Reinforcement learning (RL) is one of the best techniques to make optimal decisions, and many studies have recently been conducted on this topic [18,19,20]. Alike the method using the heuristic optimization algorithm [21,22], the newly proposed feature selection methods using RL operate flexibly on any dataset and can be adjusted for each feature, enabling a detailed judgment. Feature selection methods using RL, such as the heuristic optimization algorithm, work well for multiple datasets but they are inefficient because they require significant computation time and computing power to search many states [23].

The objective of this study was to develop an effective and efficient feature selection method using RL algorithms. If no additional algorithm advice is provided or there are no criteria to evaluate the agent’s own behavior [24,25,26], the agent is unaware of the purpose of the problem to be solved, and there is a high probability that the user will behave unexpectedly. Therefore, we proposed an agent, called a guide agent, and designed it to evaluate its own behavior. Due to the fact that these guide agents randomly set their behavior, they can be flexibly operated on any dataset, unlike external algorithms. Our proposed feature selection algorithm selects features that are effective for classification in most datasets. In reinforcement learning, where the criterion for giving a reward is very important, by introducing a method called Guide Agent, Main Agents, who play a role in actual feature selection, effectively select features through repetitive learning and experience. Existing feature selection methods also performed well on most data sets, but the proposed algorithm showed surprisingly better results, especially on data sets where specific relationships are difficult to determine, such as the colorectal cancer data set.

**Figure 1:**
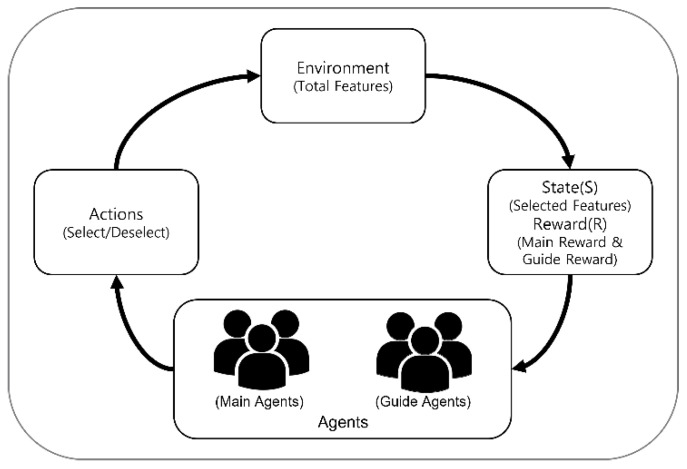
Illustration of Multi-Agent Reinforcement Learning Feature Selection Method with Guide Agents (MARFS-GA).

Figure 1 shows the flow of the multi-agent reinforcement learning feature selection method using a guide agent (MARFS-GA) proposed in this study. For a given dataset, two groups of agents called the main agents and the guide agents perform their respective actions to select features. Two rewards are calculated from the selected features using a classification algorithm. The algorithm proposed in this study uses these two rewards to obtain a learning reward and trains the main agent responsible for feature selection. Using the proposed algorithm, experiments were conducted on six datasets, and the superiority of the proposed feature selection method was verified through experimental results.

## 2. Related Works

In 2019, Liu proposed a multi-agent reinforcement learning framework for the feature selection problem. In this framework, by assigning an agent to each feature, feature selection is performed using an RL framework. Then, the state is obtained using a statistical description, autoencoder, and graph convolutional network (GCN) for the selected feature subset, so that the algorithm can sufficiently understand the learning progress. It achieved the highest accuracy of 0.8731 for the forest cover type dataset using the above framework [27]. In 2020, Fan et al. proposed an algorithm that balances efficiency and effectiveness by combining feature selection methods using RL and other feature selection methods, such as K-best, decision-tree-based ranking, and mRMR. For each agent of the RL feature selection algorithm, the aforementioned K-best, decision tree-based ranking, and mRMR are designated as external trainers to provide advice to the agents. Using this method, they achieved accuracies of 0.80~0.81, 0.93, 0.91, and 0.98 for the forest cover type, Spambase, insurance company benchmark, and Musk datasets, respectively [28]. A paper published by Khurana et al. in 2018 presented a framework using RL to automate feature engineering. They presented a method for the exploration of a transformation graph that systematically and compressively captures the space of a given option. After listing a subset of a given dataset through a transformation graph, the agent explores the space of the listed feature choices. This method demonstrated an accuracy of 0.961 for the Spambase dataset [29]. A paper published by Rasoul et al. in 2021 formalized the state space as a Markov decision process and used the temporal difference algorithm to find a subset of the best features. It achieved an accuracy of 0.7629 on the breast cancer Wisconsin dataset [30]. Finally, the method presented by Fard et al. in 2013 considers the state space of a feature as a Markov decision process and then proceeds with an optimal graph search. Each state is evaluated using the lattice of the feature sets. Finally, the optimal features are selected using a method based on filters and wrappers. This method demonstrated an accuracy of 0.7306 for the colon cancer dataset [31].

## 3. Methods

### 3.1. Definitions

Agents (Agt): One agent is assigned to each feature. For example, assuming 30 features, 30 agents are created. The agents assigned to each feature determine whether to select a feature. There are two types of agents: main and guide agents.

Main Agents (Agt_m_): The main agents are agents that perform feature selection. They perform the main action and learn by receiving a reward based on the action.

Guide Agents (Agt_g_): Guide agents are those that are not involved in feature selection. They do not learn even if they receive a reward for their actions, and all their actions are randomly determined.

Action (A): Each agent decides whether to select a feature. The action of the i^th^ agent A_i_ is either zero or one. Zero means that the feature is not selected, and one means that the feature is selected. In this study, there are two types of actions: the main action, which is the action performed by the main agents, and the guide action, which is performed by the guide agents.

Main Actions (A_m_): The main action is performed by the main agents. It is an indicator for selecting a feature and has a Q-value that indicates the value of each action.

Guide Actions (A_g_): Guide actions are performed by guide agents. Guide behavior is an index for evaluating whether the main agents are correct when performing the main behavior. Unlike the main action, a guide action does not have a Q-value that indicates the value of each action.

Environment (E): Environment refers to the environment in which the agents operate. In this study, the entire dataset is defined as the environment.

State (S): In RL, the state refers to the situation after the agents have performed actions in the environment. In this study, the state is defined as a subset of features selected based on the actions of the main agents for each episode.

Reward (R): The reward is based on the actions of the agents. In the case of classification problems, the reward is defined as the classification accuracy of the selected features. In this experiment, there were three types of rewards: main, guide, and learning.

Main Reward (R_m_): This is the reward for the characteristics selected through the main actions performed by the main agents. It is defined as the classification accuracy of the features selected through the main actions. It is not used for learning the value of the main agents’ actions.

Guide Reward (R_g_): This is a reward for the characteristics selected through guide actions performed by the guide agents. It is defined as the classification accuracy of the features selected through the guide actions. Alike the main reward, it is not used as a reward for learning the value of the main agents’ actions.

Train Reward (R_t_): This reward is used to learn the values of the main agents’ actions. The learning reward is defined as the difference between the main and guide rewards.

### 3.2. Experiments

**Figure 2:**
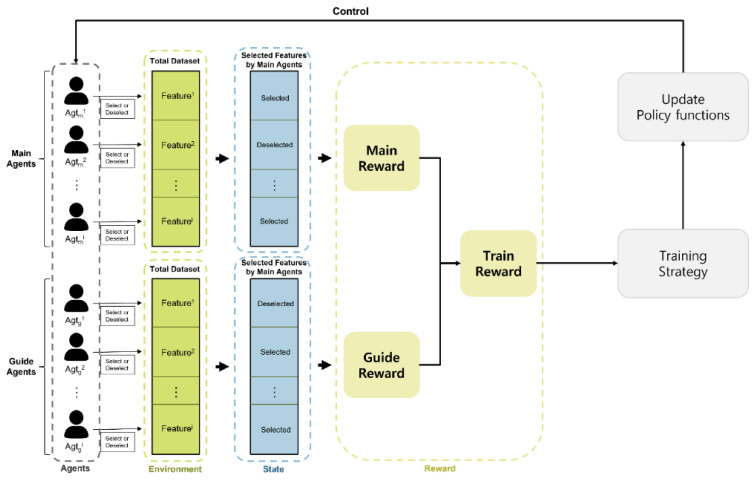
Framework Overview.

Framework overview: Figure 2 shows the overall framework of the experiment. Main agents and guide agents each perform an action. The Main Action and Guide Action performed by them interact with the environment corresponding to the dataset. Each agent acquires a reward (main reward and guide reward) according to the state after interacting with the environment through the Action. Then, the train reward is calculated using the derived reward, and the behavior (Q-value) of the Main Agents selected by the training strategy is learned as the train reward value.

**Figure 3:**
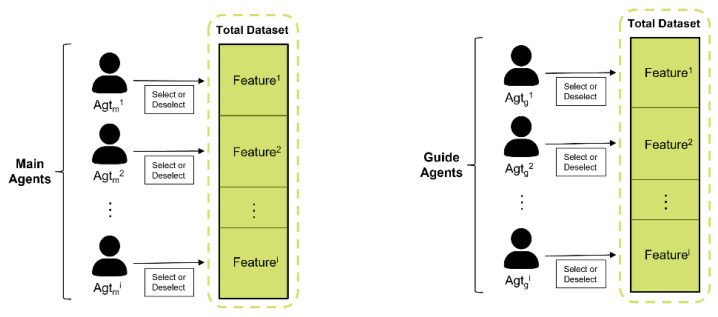
Initializing Main Agents and Guide Agents.

Initializing Main and Guide Agents: Figure 3 illustrates the creation of the main and guide agents. When the algorithm starts, the number of main and guide agents created by each is equal to the number of features in the data set. For example, if there are 30 features in one dataset, 30 main agents and 30 guide agents are created. The agents in each group perform actions on the same dataset. The main agent performs feature selection, and the guide agent is used by the main agent to evaluate its own behavior. Therefore, the main agents have a Q-value indicating the value of their actions, and the guide agents do not have a Q-value because they are only evaluation targets.

Actions of main agents: When the episode starts, the main agents decide their actions according to the epsilon-greedy policy. This policy conducts exploration with epsilon (ε) probability based on the epsilon (ε) value and exploits it with 1−epsilon (ε) probability [32]. Here, the epsilon (ε) value has a real value between 0 and 1. Further exploration shows that the behavior of the main agent (0 or 1) is determined with half-and-half probability. In the case of exploitation, the actions are selected based on the Q-value of each main agent. The Q-value value indicates how good each action is when the action is performed, and it exists for each of the two actions (Select: 1/Deselect: 0) that each main agent can take. The agent performs the action with the larger Q-value. In this case, the action of the ith main agent can be expressed as follows: $A_{m}^{i}$ is the behavior of the ith main agent, and x ~ Uniform (0, 1) is a random real value between 0 and 1. Q(1) and Q(0) are the Q-values of the feature-selecting and non-selecting actions, respectively.

(1) $$A_{m}^{i}=\{\begin{array}{c} 0\;\mathrm{or}\;1,\;\;\;\;\;\;\;\;\;\;\;\;\;\;\;\;\;\;\;\;\;\;\;\;\;\;\;\;\;\;\;\;\;\;\;\;\;\;\;\;\;\;\;\;\;\;\;\;\;\;\;\;\;\;\;\;\;\mathrm{if}\;\varepsilon >x\;\sim \;\mathrm{Uniform}\left(0,\;1\right)\\ \{\begin{array}{c} 1,\;\;\;\;\;\;\;\;\mathrm{if}\;Q\left(1\right)\;\geq \;Q\left(0\right)\\ 0,\;\;\;\;\;\;\;\;\;\;\;\;\;\;\mathrm{if}\;\left(1\right)<\left(0\right) \end{array}\;\;\;\;\;\;\;\;\;\;\;\;\;\;\;\;\;\;\;\;\;\;\;\;\;\;\mathrm{if}\;\varepsilon \;\leq \;\;x\;\sim \;\mathrm{Uniform}\left(0,\;1\right) \end{array}$$

When one episode ends, the epsilon value is reduced by the epsilon decay rate (EDR) value. The EDR has a real value between 0 and 1. Thus, the ratio of utilization between exploration and exploitation gradually increases. This is expressed as:

(2) ε = ε × EDR

Actions of guide agents: When the episode starts, guide agents, unlike the main agents, randomly (with a half chance) determine all their actions. There is no Q-value for the actions of the guide agents, and the actions performed at time t do not have any effect on the actions performed at time t+1. The action of the ith guide agent is expressed as follows:

(3) $$A_{g}^{i}=0\;\mathrm{or}\;1$$

Reward calculation method: The main and guide rewards are defined as the accuracy of features selected through the main actions performed by the main agents and guide actions performed by the guide agents, respectively. The learning reward is defined as the difference between the main and guide rewards; when the main reward is higher, it becomes a positive reward, and when the guide reward is higher, it becomes a negative reward. The reward calculation method is expressed as follows:

(4) $$R_{t}={\;R}_{m}-{\;R}_{g}$$

where R_t_ is the learning reward used for learning the values of the main agent, R_m_ is the main reward calculated by the action of the main agent, and R_g_ is the guide reward calculated by the action of the guide agent. Since R_m_ and R_g_ are classification accuracies with features selected by the main agents and guide agents as input values, both values have a value between 0 and 1. Therefore, R_t_ has a minimum value of 0 and a maximum value of 1.

**Figure 4:**
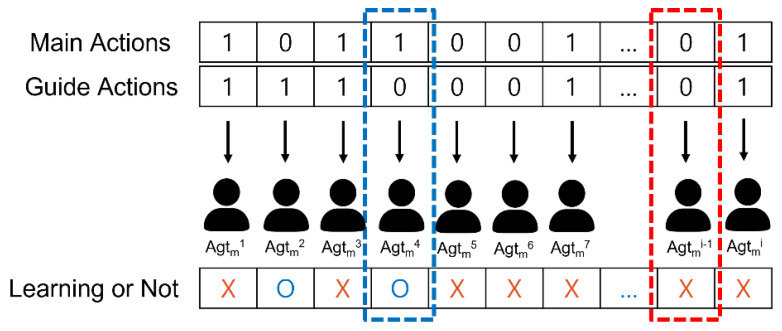
Training Strategy.

Training strategy for updating the Q-value of main agents: After one episode, not all the main agents learn. If the main action is performed by the main agent at the same location it is in, and the guide actions performed by the guide agent are different from each other, the main agent corresponding to the location learns. For example, if the third main and guide actions are the same, the third main agent is not rewarded with a train reward. Conversely, if the fourth main and guide actions are different from each other, the fourth main agent will be rewarded with the train reward for the action it took. Figure 4 shows a schematic of the training strategy.

There are two reasons for learning when different actions are performed. First, the difference between the accuracies of the main and guide actions is that different actions occur at the same location; hence, only the agent at that location learns. Second, if a train reward is given to all main agents, detailed adjustment of the main agents’ actions becomes impossible. This is because if the accuracy of the main action continues to be higher than that of the guide action, all main agents will continue to receive rewards as they cannot filter out less important features such as noise or noise.

When the main agents that receive the reward are determined through the above process, each main agent multiplies the Q-value of the action they execute and adds them to the learning rate (α) value. At this time, the α value is responsible for adjusting the amount of train reward reflected and has a real value between 0 and 1. For the action of the i^th^ main agent, the Q-value updates expression through the train reward, which can be expressed as:

(5) $$Q\left(A_{m}^{i}\right)=\;Q\left(A_{m}^{i}\right)+\left(\alpha \;\times {\;R}_{t}\right)$$

where $Q\left(A_{m}^{i}\right)$ is the Q-value for the action of the ith main agent, α is the learning rate used to designate the degree of learning, and $R_{t}$ is the reward for the action of the main agent.

The way the model operates according to the above method is as follows:

(1) Guide agents and main agents are created according to the number of features in the data set.
(2) According to our proposed method, features to be used for classification are selected.
(3) Based on the selected features, the data set is divided at a ratio of 7:3 and used as the training data and test data of the classifier.
(4) The test result (accuracy) is used as a reward to improve the performance of the model.
(5) Repeat the above process as many times as the number of episodes you specified, or until you reach the target you want.
(6) When the process up to step 5 is completed, the features finally selected by our proposed methodology are judged to be suitable for classification.

### 3.3. Algorithm

The above process is expressed as Algorithm 1.
**Algorithm 1:** Multi-Agent Reinforcement Learning Feature Selection Method with Guide Agents (MARFS-GA).Input: Number of Features: N, Epsilon value: ε, Learning Rate: α, Number of Episodes: E, Epsilon Decay Ratefor i = 1 to N do # make Agents    make Agt_m_^i^, Agt_g_^i^End forfor i = 1 to N do # initialize Q-values    Q-value of Agt_m_^i^ [random value, random value]End forfor E = 1 to Number of Episode do    for i = 1 to N do        if random value > ε do           Agt_m_^i^ do Exploitation        else do           Agt_m_^i^ do Exploration    End for    for i = 1 to N do        Agt_g_^i^ do Random Action    End for    Selected Features by A_m_ = Select Features (A_m_)    Selected Features by A_g_ = Select Features (A_g_)    R_m_ = get Reward (Selected Features by A_m_)    R_g_ = get Reward (Selected Features by A_g_)    R_t_ = R_m_ − R_g_ # Classification Problem    for i = 1 to N do        if A_m_^i^ ≠ A_g_^i^            Q-value of A_m_^i^ + = α × R_t_    End for    ε = ε × Epsilon Decay RateEnd forReturn Selected Feature Subset

## 4. Results

### 4.1. Datasets

Breast cancer Wisconsin: This is a breast cancer dataset provided by the University of Wisconsin comprising 212 negative samples and 357 positive samples, for a total of 569. The total number of features is 32, including the radius, texture, and perimeter [33].

Forest cover type: This dataset categorizes seven forest cover types and consists of 581,012 samples. It has 54 features including elevation, quantitative, slope, and soil types [33].

Spambase: This is a short message service (SMS) dataset with a label indicating whether a message is spam or non-spam. It contains 4601 samples, consisting of 1813 Spam messages and 2788 non-spam messages. There are 57 features in this dataset, and each feature consists of information about the number of specific characters appearing in a message [33].

The insurance company benchmark: This dataset is labeled according to whether a customer has purchased Caravan insurance. There are 9822 samples, consisting of 586 subscriber data and 9236 non-subscriber data. This dataset has 86 features, which are composed of product usage data and socio-demographic data derived from zip-area codes [33].

Musk: This is a dataset with a Musk or non-Musk label for each data. It has 6598 samples that consist of 1017 Musk data and 5581 non-Musk data and 168 features. The first two attributes of the Musk dataset were excluded from this experiment because they refer to the names of molecules and forms [33].

Colon cancer: This is a dataset provided by Princeton University. It has 62 samples of which 40 contain normal data and 22 contain colorectal cancer patient data. It has 2000 features, and each feature represents gene information [34].

### 4.2. Experiment Settings

The composition of the variables in this experiment was as follows: The initial epsilon (ε) value was 0.5, and the EDR was 0.99995. The number of main and guide agents generated depends on the number of features in the dataset being tested. The learning rate was 0.01 and the maximum number of episodes was 10,000. The reason for setting the EDR to 0.99995 is that if the EDR is 0.99995 when the initial epsilon value is 0.5, the epsilon value of the last step will drop to about 0.3 if 10,000 episodes are performed. If you run 1000 episodes, setting the EDR to 0.9995 will reduce the epsilon value to about 0.3 in the last step. For the initial main agent action, the Q-value corresponding to 0 (Deselect) was randomly initialized to a real value between 0 and 1, and the Q-value corresponding to 1 (Select) was randomly initialized to a real value between 0 and 0.05. This was intended to allow a small number of features to be initially selected, and to gradually increase the number.

In this experiment, an artificial neural network was used as the classifier. It consists of an input layer, two hidden layers, and an output layer. The two hidden layers consist of five and two nodes, respectively. Furthermore, the rectified linear unit function was used as the activation function and the learning rate was set to 0.01. Epochs were assigned a maximum score of 10. Prior to the experiment, the training data and test data were configured in a ratio of 7:3. The artificial neural network was trained with training data corresponding to the features selected by the main agent for each episode and evaluated with test data.

In addition, to compare and verify the performance of the proposed method, feature selection was performed using the mRMR, Relief, and genetic algorithm. They were implemented and directly tested. In the case of the genetic algorithm, only a basic concept was used without introducing a special method, and the fitness value of the genetic algorithm was defined as the classification accuracy. In this case, a classifier for obtaining classification accuracy had the same configuration as that of the aforementioned classifier.

### 4.3. Results

**Figure 5:**
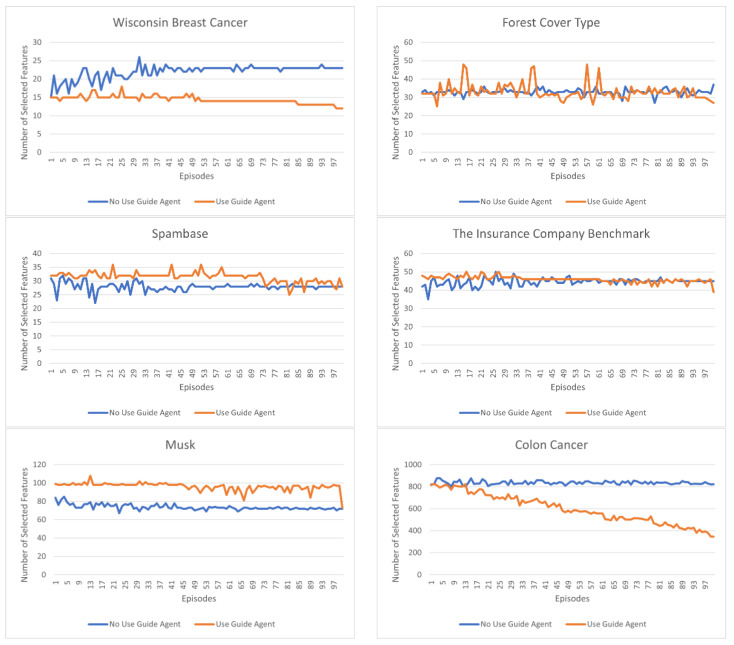
Number of selected features per episode, for each dataset.

**Figure 6:**
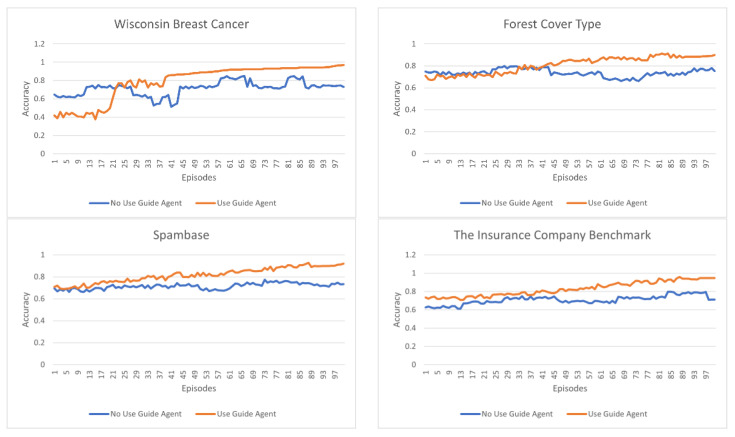

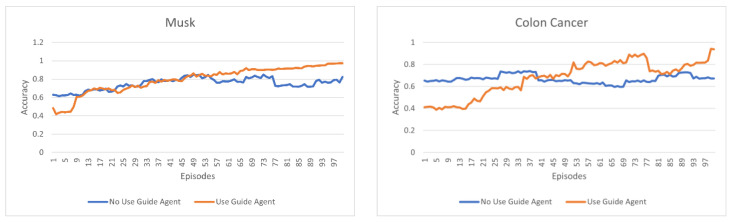
Accuracy of selected features per episode, for each dataset.

In Figure 5 and Figure 6, the orange color represents the results of the model using the guide agent, and the blue color represents the results of the model without the guide agent. In the model that does not use the guide agent, only the main agent exists, and all agents receive the accuracy obtained according to the actions of the main agent as a reward for their actions. The configuration of the model not using the guide agent is the same as the configuration of the model using the guide agent.

Figure 5 shows the number of features selected for each episode in each dataset, and Figure 6 shows the classification accuracy for each episode. For each experiment, 10,000 episodes were performed, and the values plotted on the graph are the average values of the number and accuracy of the features derived per 100 episodes.

It is evident that the experiments conducted with the guide agent increase the classification accuracy compared with the experiments that do not. In the experiments wherein the guide agent is applied, the classification accuracy increases as the episode progress, but in those without the guide agent, there is either no significant increase compared to the initially derived accuracy, or the accuracy is constant. It is not always maintained and appears to rise or fall continuously. In the experiments that did not apply the guide agent, all agents received the initial result values as a reward without any strategy. Therefore, it is evident that this is because, in the beginning, the Q-value for one action increases considerably and the opportunity to take another action decreases. In addition, the continuous change in accuracy can be attributed to a behavioral method of agents, called exploration. Exploration causes an agent to perform various actions, and it appears that the change in accuracy is significantly caused by such an exploration action. Therefore, the accuracy of the selected characteristics can be seen to vary greatly when there are significant changes in the types of selected characteristics because this greatly affects the change in the behavior of the entire agent.

Through various experiments, it was deduced that the method in which the guide agent is applied does not change the number of selected features and the accuracy constantly increases. By applying the guide agent, the main agent can reliably determine whether their actions are correct, and by learning only a small number of features through the proposed learning strategy, large fluctuations can be avoided. However, in the case of the forest cover type dataset, the selection number of features changes significantly compared to other datasets. This is when many of the features of the forest cover type dataset have meaningless values. These features are filtered out during the learning process, while other features are selected; hence, the amount of change is large compared to other datasets.

Table 1 lists the experimental results for each dataset, where each numerical value represents the classification accuracy. The numbers on the left in parentheses indicate the total number of features in the dataset, and the numbers on the right indicate the number of features finally selected by applying our algorithm. Evidently, compared with other experiments, our proposed method achieves a slightly better performance. In other methods, there were no data on the number of selected features; therefore, a comparison of the number of features could not be made. The results of six experiments show that our proposed feature selection method enables efficient feature selection for classification, regardless of the number of features in the dataset. Table 2 lists the results of our proposed method, and the feature selection methods commonly used in other studies. The results obtained after applying each feature selection method are that of the features showing the greatest accuracy. Table 1 and Table 2 refer to the average value of the accuracy obtained by performing a total of 10 experiments for each dataset.

In the case of the Wisconsin breast cancer dataset, there was no significant difference in accuracy between the four feature selection methods and our proposed method; however, the mRMR method achieved the highest accuracy. A satisfactory result of 0.9404 was obtained even when the experiment was conducted without feature selection. All features of the Wisconsin breast cancer dataset have some significant characteristics. Moreover, from the results in Table 2, it can be observed that the linear data analysis method is the most efficient for this dataset.

For the forest cover type dataset, our algorithm achieved the best result, with an accuracy of 0.8802. In the case of this dataset, the accuracy improved by approximately 0.17 or more when the feature selection method was used. However, it is judged that the linear analysis method, that is, the selection method based on a combination of features, does not show very good performance because the improved classification accuracy is not very high.

For the Spambase dataset, our algorithm achieved the best results, with an accuracy of 0.963. Compared with other experimental results, the Genetic Algorithm and proposed method achieved high accuracy. From the results of these experiments, in the case of this dataset, the feature selection method based on a combination of features achieved better results than the linear analysis method.

In the case of the insurance company benchmark dataset, our algorithm achieved the second-highest result with an accuracy of 0.943. The feature selection method using Relief achieved the best accuracy. A similar method, mRMR, also achieved high accuracy. The genetic algorithm’s accuracy was relatively low. Therefore, in the case of the insurance company benchmark dataset, there seems to be a linear relationship between features and labels.

For the Musk dataset, the proposed method achieved the highest accuracy of 0.984. Compared with when no method was used, it achieved a significant accuracy improvement when feature selection was performed. It can be observed that the Musk dataset contains many meaningless features. Compared with the other algorithms, the genetic algorithm and our proposed method achieved higher classification accuracy. However, in the case of the experiment using mRMR, it can be assumed that there is linearity between each characteristic and the label of the Musk dataset, as it shows high accuracy.

The experiment with the colon cancer dataset showed the greatest difference compared to the other dataset experiments. Feature selection using the linear analysis method achieved slightly higher accuracy than when no method was used. However, the feature selection methods based on combinations, such as the genetic algorithm and our algorithm, showed superior performance. In addition, the difference in the results obtained using our proposed feature selection method and the genetic algorithm was 0.0364; a difference of approximately 3.64%.

Models that do not use guide agents converge at a fast rate, either in terms of accuracy or in the number of selected features and are less likely to go in the right direction, even with incorrect results. On the other hand, the model using the guide agent tends to improve gradually even if the accuracy or the number of selected features converge to some extent in the beginning. This is because the main agent refers to the characteristics and accuracy selected by the guide agent, which acts as a guide for the main agent’s behavior and acts. Since the main agents are rewarded through the difference in accuracy between the main agent and the guide agent, the main agent can know whether their behavior is good or bad compared to the behavior of the guide agent.

## 5. Conclusions

Our proposed feature selection algorithm selects features that are effective for classification in most datasets. Our proposed model initially selects features randomly and then selects features according to the Q value of the main agent. The main agent sees the behavior of the guide agent, judges whether their behavior is correct, and determines whether it is better to change the decision on feature selection or keep it by comparing it with the current state according to the reward and Q value. Even if the accuracy for the result of not selecting a feature is high, if the Q value for the behavior of selecting a feature is high, the feature is selected as it is. Existing feature selection methods also performed well for most datasets, but our proposed algorithm showed surprisingly better results, especially for datasets where it is difficult to determine a specific relationship, such as the colon cancer dataset. Since the results in Table 1 are the results of the highest accuracy published in each paper, the classifier or the ratio of dividing the data set does not match the experiment in this paper. Therefore, although an exact comparison is not possible, this paper arguably has sufficiently demonstrated the possibility because it showed excellent performance by using the most basic classifier, an artificial neural network. Widely used feature selection methods, such as mRMR and Relief, define the relationship between data by using formulas. Therefore, it is difficult to apply them to data that show high performance when several features are applied together, although the relationship between them is weak. In addition, the feature extraction method using the genetic algorithm, which is used as an alternative, is similar to the direction we are pursuing but has a limitation in that it focuses on the combination of features. In this study, the proposed algorithm can learn more flexibly by learning each feature individually, or a combination of features. To design the method proposed in this paper, we focused on emphasizing individual features rather than focusing on a combination of features. Since it is tailored, a detailed selection is not performed well. Although most of the same features were selected whenever the experiment was repeated, problems such as features mixed with noise continued to occur. In addition, although all guide agents’ actions are randomly assigned, only two types of actions can be taken. Therefore, if one action occurs continuously, there is a high possibility that the action of the main agent corresponding to that location will be concentrated on one side. Therefore, in the future, we plan to develop a learning method for each agent to select features in more detail.

## Figures and Tables

**Table 1:** Experiment results for each dataset using the proposed method and other RL methods.

Dataset	Proposed Method	2019, Kunpeng Liu	2020, Wei Fan	2018, Udayan Khurana	2021, Sali Rasoul	2013, Seyed Mehdi Hazrati Fard
Wisconsin Breast Cancer	0.982 (32→17)	-	-	-	0.7629	-
Forest Cover Type	0.8802 (54→33)	0.8731	0.80~0.81	-	-	-
Spambase	0.963 (57→20)	-	0.93	0.961	-	0.8293
The Insurance Company Benchmark	0.943 (86→27)	-	0.91	-	-	-
Musk	0.984 (166→30)	-	0.98	-	-	-
Colon Cancer	0.947 (2000→38)	-	-	-	-	0.7306

**Table 2:** Experiment results for each dataset using the proposed method and other feature selection algorithms.

Dataset	Proposed Method	mRMR	Relief	Genetic Algorithm	No Use
Wisconsin Breast Cancer	0.982 (32→17)	0.9867	0.9706	0.9702	0.9404
Forest Cover Type	0.8802 (54→33)	0.696	0.684	0.6315	0.5279
Spambase	0.963 (57→20)	0.8608	0.8595	0.9168	0.8407
The Insurance Company Benchmark	0.943 (86→27)	0.9343	0.9531	0.9012	0.644
Musk	0.984 (166→30)	0.9256	0.9012	0.9312	0.8439
Colon Cancer	0.947 (2000→38)	0.861	0.8806	0.9106	0.7419

## Data Availability

The dataset mentioned in this study can be found at http://archive.ics.uci.edu/ml and http://genomics-pubs.princeton.edu/oncology (accessed on 1 September 2020).
